# Modulating effects of *Phellinus linteus* polysaccharides on antioxidant capacity, immune function, intestinal function and microbiota in lipopolysaccharide-challenged broilers

**DOI:** 10.3389/fmicb.2025.1570370

**Published:** 2025-05-27

**Authors:** Yang Li, Shuai Wang, Xuran Zhu, Nana Gao, Jungang Kang, Tianxiong Wang, Xiaodan Wang

**Affiliations:** ^1^College of Traditional Chinese Veterinary Medicine, Hebei Agricultural University, Baoding, China; ^2^Chengde Academy of Agricultural and Forestry Sciences, Chengde, Hebei, China

**Keywords:** *Phellinus linteus* polysaccharides, antioxidant, broiler, lipopolysaccharide, microbiota

## Abstract

*Phellinus linteus* polysaccharides (PLP) have been shown to have beneficial effects on gut function and gut microbiota in animals. The intention of this study was to examine the effects of PLP on growth performance, immune function, intestinal barrier function and intestinal microbial community in broilers under lipopolysaccharide (LPS) challenge. A total of 120 one-day-old Arbor Acres (AA) broilers were randomly allocated into 3 groups: control, LPS, and PLP + LPS. The PLP + LPS group had 200 mg/kg/d PLP added to the ration in the daily trial. LPS and PLP + LPS group were intraperitoneally injected with LPS on days 14, 16, 18, and 20. The results demonstrated that LPS significantly decreased average daily gain (ADG), average daily feed intake (ADFI), and total antioxidant capacity (T-AOC) activity in serum and duodenum. Additionally, LPS reduced the mRNA expression levels of *ZO-1* and *Occludin* in duodenum, increased spleen bursal index, and MDA activity in serum and duodenum (*P* < 0.05). Histological examination revealed that LPS caused duodenal damage, leading to intestinal mucosal injury and shedding, villus height reduction, and crypt depth increase. PLP inclusion mitigated the adverse impacts of LPS on key parameters (*P* < 0.05). Furthermore, gut microbiota analysis revealed that PLP supplementation increased the ratio of *Firmicutes* to *Bacteroidetes*. At the genus level, the addition of PLP decreased the abundance of *Bacteroides* and *Escherichia-Shigella*, while simultaneously increased the presence of *Akkermansia, Faecalibacterium, Lactobacillus*, and *Parabacteroides*. In summary, supplementing the dietary inclusion with 200 mg/kg of PLP enhances growth, immune function, and antioxidant capabilities in LPS-challenged broilers. This improvement is likely attributed to the modulation of gut microbiota community composition.

## Graphical Abstract

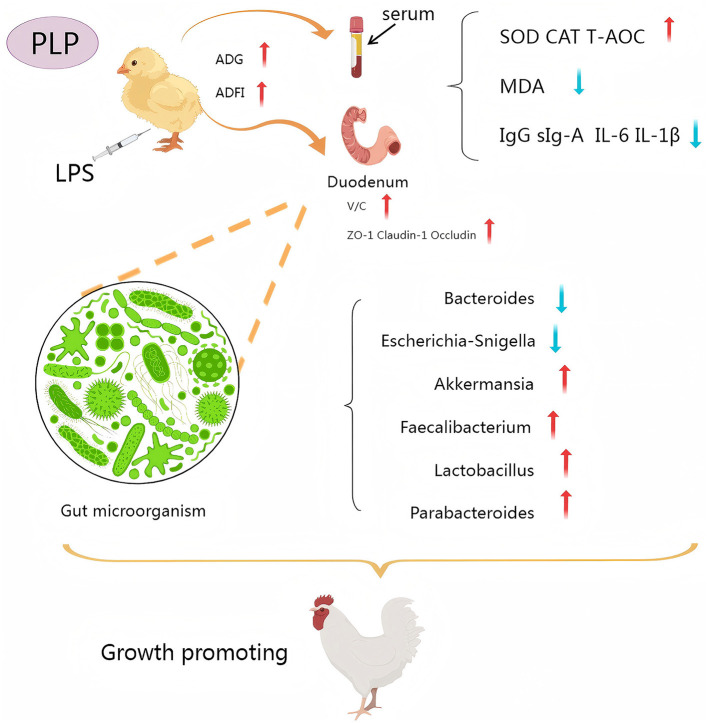

## 1 Introduction

With the global intensification and expansion of poultry farming, farms are increasingly vulnerable to outbreaks of pathogenic bacterial infections and environmental contamination (Xi et al., 2023). In the next decade, the growth rate of poultry consumption is expected to exceed the total global meat consumption growth by more than 50% (Harchaoui et al., 2023), intensifying pressure on disease management. Poultry are highly susceptible to stressors such as bacterial infections and toxins, which trigger immune responses that impair growth performance, damage intestinal integrity, and increase mortality (Wang et al., 2020; Bi et al., 2022; Hu et al., 2024). Moreover, during early post-hatch development when the avian immune system is still immature, exposure to immunological stressors can suppress immune function (Song et al., 2021). To address these challenges, natural immunomodulators, particularly plant polysaccharides, have emerged as a research focus for antibiotic alternatives in poultry feed due to their sustainable advantages (Du et al., 2023; Guo et al., 2024).

PLP is derived from *Phellinus linteus*, with a long history in China, has a variety of biological activities, including antibacterial, antioxidant and immunomodulatory effects, as well as liver protection and hypoglycemic properties (Xie Z. et al., 2019; Qin et al., 2023). Studies have found that PLP inhibits the translocation of NF-κB and increases the phosphorylation of AMPKα, thereby reducing the pro-inflammatory cytokines in RAW264.7 cells and increasing the anti-inflammatory cytokines (Liu J. et al., 2023). In addition to its anti-inflammatory effects, it is also crucial for regulating the composition and abundance of gut microbiota (Zapora et al., 2016; Wang et al., 2019). PLP can be used to improve intestinal ecological balance and preserve intestinal barrier function by enhancing the concentration of short-chain fatty acid in diabetic rats' gut (Liu et al., 2020). LPS, a potent immune activator derived from Gram-negative bacteria, plays a pivotal role in triggering immune responses (He et al., 2022; Hu et al., 2023).

Most existing studies on PLP have focused on mammalian models, while the effects of PLP on the intestinal tract of poultry have not been reported. Therefore, this study aims to investigate the effects of PLP on LPS-induced growth performance, antioxidant capacity, immunomodulatory responses, intestinal morphology, and duodenal microbiota in broiler chickens by establishing an LPS-induced immune stress model. Additionally, the protective mechanism of PLP will be further analyzed using 16S rRNA. This research aims to provide a theoretical and practical basis for the application of PLP in poultry breeding practices.

## 2 Materials and methods

### 2.1 Materials and reagents

All male broilers purchased from Tianjin Hongteng Co., Ltd. (Tianjin, China). PLP (≥50%) was provided by Shanxi Yuan Beibei Bio-Technology Co., Ltd. (Shanxi, China). LPS (Escherichia coli Serotype O55:B5) was purchased from Sigma-Aldrich (St. Louis, MO, USA). Superoxide dismutase (SOD), T-AOC, Malondialdehyde (MDA), Glutathione Peroxidase (GSH-Px) and Catalase (CAT) activity test kits were purchased from Nanjing Jiancheng Bioengineering Institute (Nanjing, China). Interleukin-1β (IL-1β), Interleukin-6 (IL-6), Interleukin-10 (IL-10) and tumor necrosis factor-α (TNF-α) kits were purchased from Shanghai Enzyme Linked Biotechnology Company (Shanghai, China).

### 2.2 Animal management and experimental design

**Figure 1:**
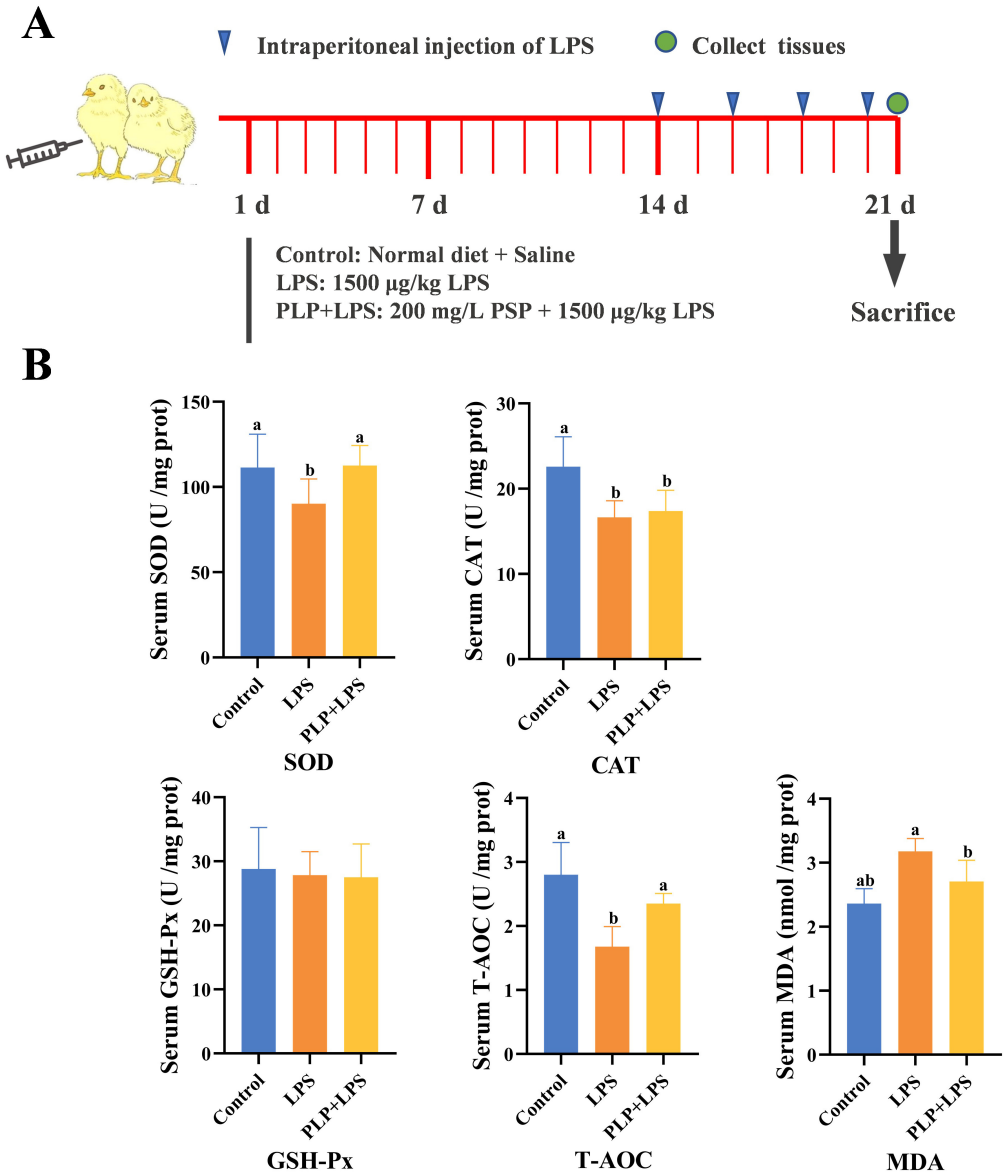
Effects of PLP on serum oxidation indices of LPS challenged broilers. **(A)** Schematic diagram of the experiment. **(B)** The same shoulder label or no letter in peer data indicates no significant difference (*P* > 0.05), different lowercase letters indicate significant difference (*P* < 0.05). Control (blank control group); LPS (1,500 μg/kg LPS challenge group); PLP + LPS (200 mg/kg PLP + 1,500 μg/kg LPS treatment group).

One hundred and twenty one-day-old broilers were randomly divided into three groups, each consisting of four replicates with ten birds per replicate. The control and LPS group were fed a basal dietary, whereas the PLP+LPS group had a dietary inclusion with 200 mg/kg PLP. LPS was administered intraperitoneally at a dose of 1,500 μg/kg body weight to broilers in the LPS group and the PLP+LPS group. The control group was injected with an equal amount of physiological saline. These injections were administered on days 14, 16, 18 and 20 of the experiment. One week before experiment initiating, disinfection and ventilation were administered to the chicken house. The temperature of the chicken house was maintained at 31-34°C in the first week. Subsequently, the temperature was gradually reduced by 2-3°C per week until reaching approximately 22°C, which was then maintained. The broilers were housed in triple cages and fed twice a day at 7:30 am and 14:30 pm. All broilers had free access to feed and water. Experimental flowchart (Figure 1A). The basal diet was formulated according to the nutritional requirements outlined in the “Chicken Feeding Standard NY/T 33-2004”. The feed formulation and nutrient levels of the basal ration are shown in Table 1.

**Table 1:** Nutrient level of experimental animal base diet formula (air-dry basis, %).

**Ingredient**	**Contents**
Corn	40.5
Soybean meal	21.65
Flour	16
Peanut meal	4
CaHPO_4_	1.11
Corn protein	6.5
Limestone	1.15
L-Lys	0.79
NaCl	0.23
Montmorillonite	0.3
DL-Met	0.12
Trace mineral premix^a^	0.08
Choline-Cl	0.08
L-Thr	0.1
Soybean oil	6.99
BV25	0.4
Total	100.00
**Nutrient levels**	
Metabolizable energy, MJ/kg	12.57
Crude protein	20.5
Crude ash	7.5
Crude Fiber	2.75
Ether Extract	3.75
Calcium	1.13
Total phosphorus	0.65
Methionine	0.56
Lysine	1.25

^*a*^Premixes provide per kilogram of ration: VA 6,141.5 IU; VD_3_ 1,681.4 IU; VE 30 IU; VK 1.91 mg; VB_1_ 9.69 mg; VB_2_ 4.01 mg; VB_6_ 1.99 mg; VB_12_ 0.01 mg; niacin 18.06 mg; calcium pantothenate 6.79 mg; folic acid 0.60 mg; biotin 0.07 mg; Fe 60.91 mg; Zn 66.75 mg; Cu 6.01 mg; Mn 68.3 mg; I 10.9 mg; Se 0.2 mg.

### 2.3 Growth performance measurement

Throughout the experiment, the health status of the broilers was meticulously monitored on a daily basis. Body weights were recorded precisely at 8:00 a.m. on both the first and twenty-first days, following a fasting period of 12 hours without food or water. Additionally, daily records were kept of both the quantity of food provided and the amount consumed. Subsequently, the ADG, ADFI and feed-to-gain ratio (F/G) were calculated for the duration of the 21-day period.

### 2.4 Sample collection

On the 21th day, the Arbor Acres broilers were humanely slaughtered by cervical dislocation. Hence, the duodenum was weighed, and blood samples were collected for further experimental. The thymus, spleen and bursa of Fabricius were collected from the broilers and weighed. After weighing, the organ index for each organ was calculated. The duodenum was isolated, and a 2 cm specimen from the mid-duodenum was placed in 4% paraformaldehyde for histologic analysis. The duodenum contents were collected and placed in 2 mL freezing tubes, which were then submerged in liquid nitrogen for rapid freezing in preparation for further 16s sequencing analysis.

Thymus index = Thymus weight (g) ÷ Body weight (g)

Spleen index = Spleen weight (g) ÷ Body weight (g)

Bursa of Fabricius index = Bursa of Fabricius weight (g) ÷ Body weight (g)

### 2.5 Determination of serum and duodenal antioxidant capacity

SOD, GSH-Px, T-AOC, CAT activity and MDA levels in serum and duodenum were measured using ELISA kits.

### 2.6 Measurement of serum and duodenal immune function

Serum and duodenal levels of IgG, sIgA, IL-6, IL-10, IL-1β and TNF-α were determined using ELISA kits.

### 2.7 Morphological analysis of the intestinal tract

Each intestinal segment was fixed in 4% paraformaldehyde (EG1150h, LEIC, Germany) within a fume hood for over 24 h. Subsequently, a series of steps including alcohol dehydration, transparency, and wax immersion were carried out. The sections were dewaxed and stained with hematoxylin and eosin (H&E). Villus height (VH) and crypt depth (CD) were then examined and analyzed using an image analyser (Image-proplus 5.0) under a light microscope.

### 2.8 Determination of *Claudin-1, Occludin* and *ZO-1* mrna expression levels in the duodenum

The mRNA expression of genes pertinent to duodenal samples collected on day 21 was assayed via quantitative real-time PCR (qRT-PCR). Subsequently, the cDNA was synthesized using HiScript III RT SuperMix for qPCR kit. The primers were designed by Dalian Bao Biological Company and their sequences are shown in Table 2.

**Table 2:** Primer sequences and primers.

**Gene**	**Primer sequence (5^′^ → 3^′^)**	**GC%**	**Tm (°C)**	**Product size/bp (bp)**
*Claudin-1*	F: 5′-CTCCCAAGCAGCTGCATATCTC-3′	54.5	63.5	171
	R: 5′-GCTCAGTCAGGCTAAGAACACCAA-3′	50.0	64.1	
*Occludin*	F: 5′-CCTTGTTGGCCATGTGCAG-3′	57.9	63.7	148
	R: 5′-GGTCCACGGTGCAGTAGTGGTA-3′	59.1	64.4	
*ZO-1*	F: 5′-TGGCAATCAACTTTGGGTAGCA-3′	45.5	64.8	156
	R: 5′-ATCCACAGAGGCAACTGAACCATA-3′	15.8	64.1	
*β-actin*	F: 5′-ATTGTCCACCGCAAATGCTTC-3′	47.6	64.4	113
	R: 5′-AAATAAAGCCATGCCAATCTCGTC-3′	41.7	64.5	

### 2.9 Duodenum microbiota

#### 2.9.1 Genomic DNA extraction

Total DNA was extracted from duodenal samples using the CTAB method, and the purity and quality of DNA were assessed by 1% agarose gel electrophoresis. An appropriate amount of DNA was then aliquoted into centrifuge tubes and diluted with sterile water to a final concentration of 1 ng/μL for subsequent PCR amplification.

#### 2.9.2 Polymerase chain reaction (PCR) amplification

The diluted genomic DNA served as the template. The V3-V4 region of the 16S rRNA gene was amplified using primers 515F (5′-GTGCCAGCMGCCGCGGTAA-3′) and 806R (5′-GGACTACHVGGGTWTCTAAT-3′). The PCR reaction was performed using Phusion^®^ High-Fidelity PCR Master Mix with GC Buffer and high-efficiency, high-fidelity enzymes from New England Biolabs to ensure amplification efficiency and accuracy. The 30 μL PCR reaction mixture contained 15 μL of 2 × Phusion Master Mix, 0.2 μL each of forward and reverse primers, 4.6 μL of ddH2O, and 10 μL of gDNA. The thermal cycling conditions were as follows: initial denaturation at 98°C for 1 min; 30 cycles of denaturation at 98°C for 10 s, annealing at 50°C for 30 s, and extension at 72°C for 30 s; and a final extension at 72°C for 5 min. PCR was performed using a Bio-Rad T100 Thermal Cycler (BIO-RAD, USA).

#### 2.9.3 Pooling and purification of PCR products

PCR products were pooled in equimolar amounts based on their concentrations and thoroughly mixed. The pooled products were then purified by 2% agarose gel electrophoresis, and target bands were excised and purified using a gel extraction kit (Qiagen, Germany).

#### 2.9.4 Library preparation and sequencing

Libraries were constructed using the TruSeq^®^ DNA PCR-Free Sample Preparation Kit. The quality and quantity of the constructed libraries were assessed using the Qubit Fluorometer, Agilent Bioanalyzer 2100 System, and quantitative PCR (Q-PCR). Qualified libraries were sequenced on the NovaSeq 6000 platform (Illumina, USA).

#### 2.9.5 Bioinformatics analysis

Operational taxonomic units (OTUs) were clustered at 97% identity, and representative sequences from each OTU were taxonomically annotated to obtain species information and abundance profiles. Alpha diversity indices (Chao1, Shannon, ACE, and Simpson) were calculated to evaluate species richness and evenness within samples. Beta diversity was assessed using principal coordinate analysis (PCoA) based on pairwise distances between samples. Linear discriminant analysis effect size (LEfSe) was performed to identify differentially abundant bacterial taxa between groups (LDA score ≥ 3). Functional prediction of microbial communities in broiler duodenal samples was conducted using PICRUSt software. Spearman's correlation analysis was performed to assess relationships between gut microbiota and other parameters.

### 2.10 Statistical analysis

The gathered experimental data were initially organized using EXCEL. Subsequently, one-way ANOVA analysis was conducted using SPSS 27.0 statistical software to analyze the entire dataset. The outcomes were reported as “mean±standard deviation”. A significance level of *P* < 0.05 was considered indicative of a statistically significant difference, whereas *P* > 0.05 indicated a non-significant difference. Graphs representing the test data were generated using Graph Pad Prism 6.0 software.

## 3 Results

### 3.1 Effects of PLP on the growth performance of LPS-induced broilers

The LPS challenge led to significant decreases in ADG and ADFI compared to the control group (Table 3; *P* < 0.05). However, dietary supplementation with 200 mg/kg PLP alleviated the negative impact on these growth indicators (Table 3; *P* > 0.05). Furthermore, the F/G ratio in the LPS group was elevated in comparison to the control group (Table 3; *P* < 0.05). Notably, there was no.significant difference in F/G between the PLP+LPS and LPS group (*P* > 0.05).

**Table 3:** Effects of PLP on growth performance of LPS challenged broilers.

**Items**	**Control**	**LPS**	**PLP + LPS**
ADG	39.06 ± 2.27^a^	35.10 ± 2.69^b^	36.84 ± 2.94^ab^
ADFI	49.76 ± 3.21^a^	46.83 ± 2.68^b^	47.78 ± 3.24^ab^
F/G	1.27 ± 0.08^a^	1.33 ± 0.04^b^	1.30 ± 0.09^b^

ADG, average daily gain; ADFI, average daily feed intake; F/G, feed to gain ratio. The same shoulder label or no letter in peer data indicates no significant difference (*P* > 0.05), different lowercase letters indicate significant difference (*P* < 0.05). Control (blank control group); LPS (1,500 μg/kg LPS challenge group); PLP + LPS (200 mg/kg PLP + 1,500 μg/kg LPS treatment group).

### 3.2 Effect of PLP on immune organ index in LPS-induced broilers

In comparison with the control group, the LPS group exhibited a statistically significant elevation in spleen index as well as bursa of Fabricius index (Table 4; *P* < 0.05). Conversely, the PLP+LPS group showed a marked decrease in Fabricius bursa index when compared to the LPS group, whereas no noteworthy variation was detected in spleen index (Table 4; *P* < 0.05). The study revealed that the inclusion of PLP attenuated the hyperplasia of the bursa of Fabricius induced by LPS stress.

**Table 4:** Effects of PLP on immune organ index of LPS challenged broilers.

**Items**	**Control**	**LPS**	**PLP + LPS**
thymus index	0.133 ± 0.142	0.198 ± 0.186	0.170 ± 0.125
spleen index	0.128 ± 0.046^b^	0.214 ± 0.066^a^	0.175 ± 0.071^a^
bursa of Fabricius index	0.188 ± 0.056^c^	0.295 ± 0.049^a^	0.230 ± 0.059^b^

The immune organ index include thymus index, spleen index and bursa of Fabricius index. The same shoulder label or no letter in peer data indicates no significant difference (*P* > 0.05), different lowercase letters indicate significant difference (*P* < 0.05). Control (blank control group); LPS (1,500 μg/kg LPS challenge group); PLP + LPS (200 mg/kg PLP + 1,500 μg/kg LPS treatment group).

### 3.3 Effect of PLP on serum oxidative indices of LPS-induced broilers

Exposure to LPS significantly decreased serum SOD activity and T-AOC levels compared to the control group (*P* < 0.05), while PLP supplementation attenuated these reductions (Figure 1B; *P* < 0.05). In contrast, PLP had no significant effect on the LPS-induced decrease in serum CAT activity (Figure 1B; *P* > 0.05). Furthermore, PLP supplementation suppressed the LPS-induced increase in serum MDA content (Figure 1B; *P* < 0.05). The experiments pointed to the fact that PLP can mitigate the LPS-induced decrease in serum SOD activity and T-AOC levels and also the increase in MDA content in broilers.

### 3.4 Effect of PLP on serum immune indices of LPS-induced broilers

**Figure 2:**
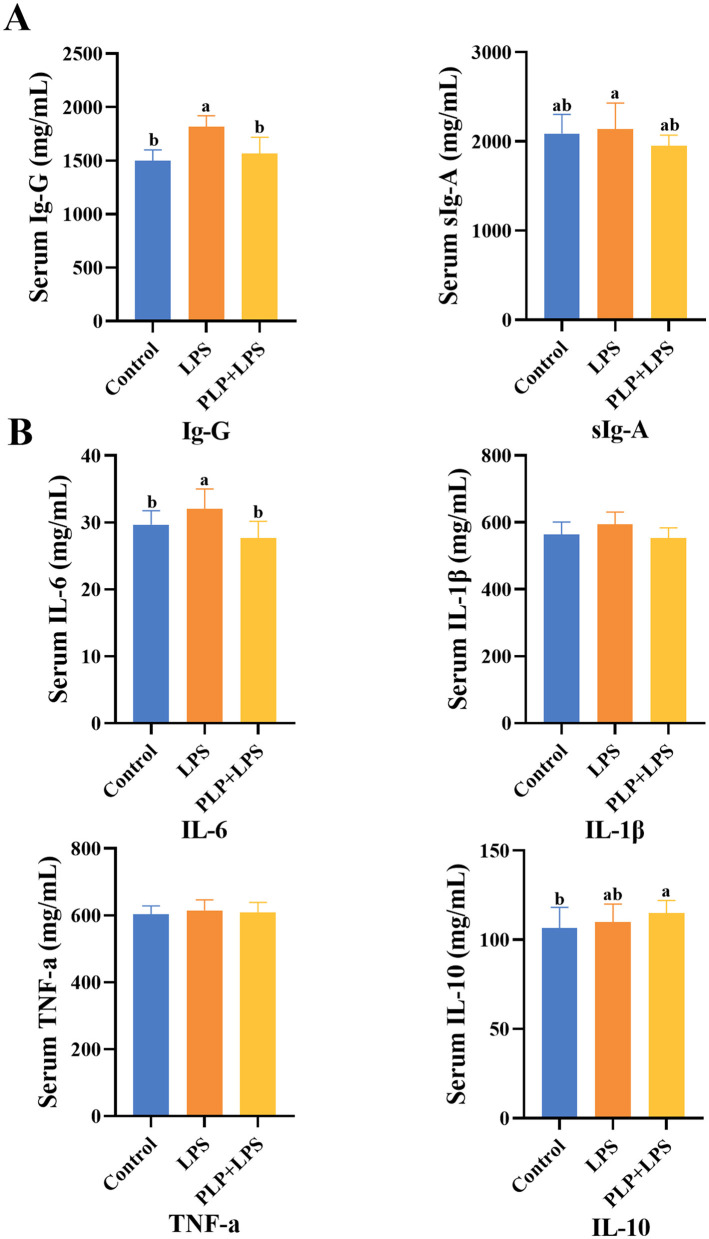
The effect of PLP on serum immune indicators and inflammatory factors of LPS challenged broilers. The effect of PLP on serum immune indicators **(A)** and inflammatory factors **(B)** of LPS challenged broilers. In the bar chart, the same superscript or no letter means no significant difference (*P* > 0.05), different lowercase letters means significant difference (*P* < 0.05). Control (blank control group); PLP (200 mg/kg PLP positive control group); LPS (1,500 μg/kg LPS challenge group); PLP + LPS (200 mg/kg PLP + 1,500 μg/kg LPS treatment group).

The serum IgG levels were significantly elevated in the LPS group compared to the control group (Figure 2A; *P* < 0.05). In contrast, the PLP+LPS group showed a marked reduction in serum IgG levels relative to the LPS group (Figure 2A; *P* < 0.05). While no significant differences in serum sIgA among treatment groups (*P* > 0.05). The findings suggest that PLP supplementation counteracts LPS-induced humoral immunity hyperactivation in broilers, as evidenced by normalized serum IgG levels.

### 3.5 Effect of PLP on serum inflammatory factors in LPS-induced broilers

Serum IL-6 levels were significantly elevated in the LPS group compared to the control group (Figure 2B; *P* < 0.05). PLP supplementation significantly attenuated this LPS-induced increase in IL-6 (*P* < 0.05). No significant differences were observed in serum levels of IL-1β, TNF-α, or IL-10 among groups (*P* > 0.05). These results indicate that PLP specifically regulate LPS-triggered immune hyperactivation by modulating IL-6 production in broilers.

### 3.6 Effect of PLP on LPS-induced oxidative indices in the duodenum of broilers

**Figure 3:**
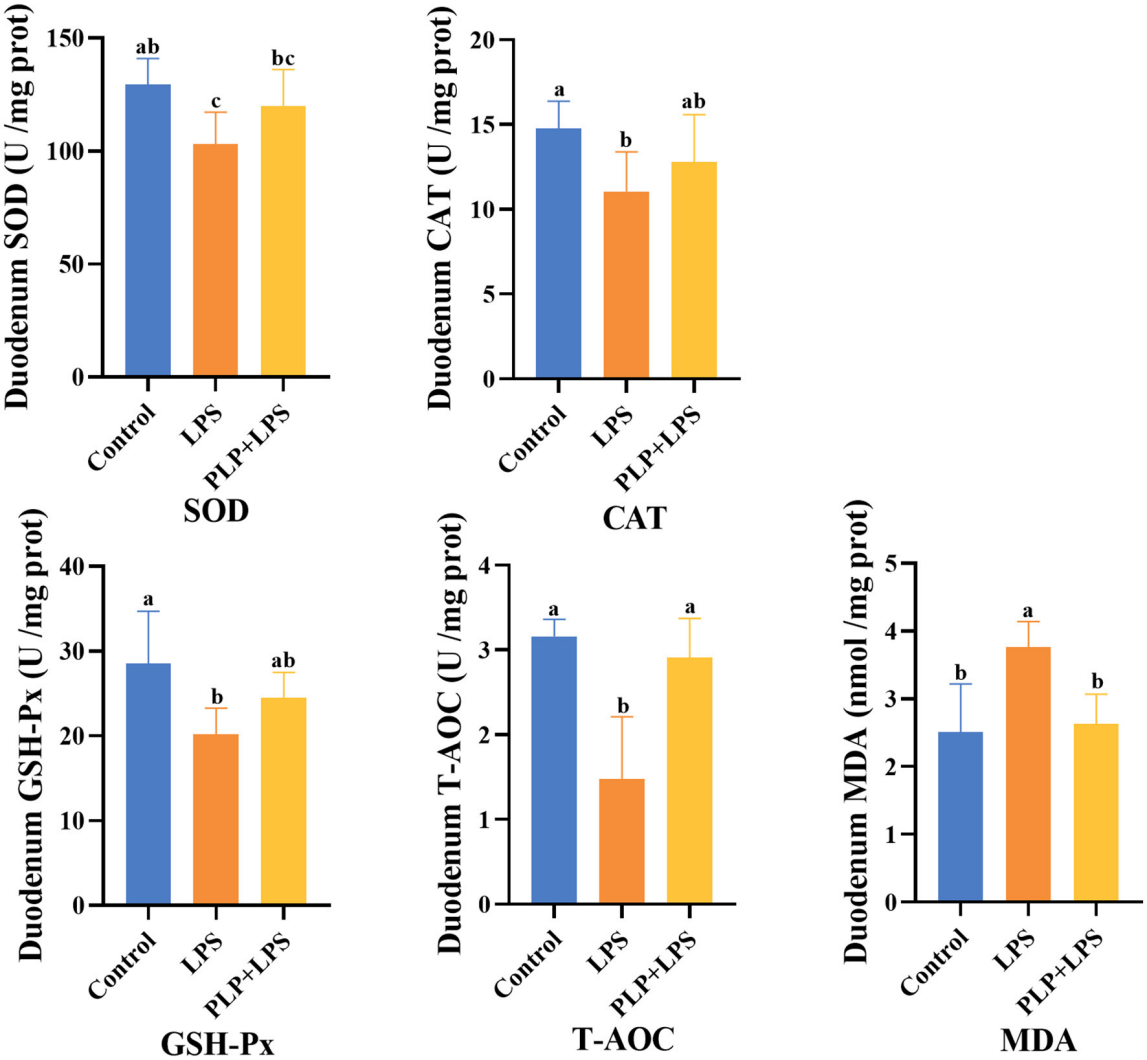
Effects of PLP on duodenum oxidation indices of LPS challenged broilers. In the bar chart, the same superscript or no letter means no significant difference (*P* > 0.05), different lowercase letters means significant difference (*P* < 0.05). Control (blank control group); LPS (1,500 μg/kg LPS challenge group); PLP+LPS (200 mg/kg PLP + 1,500 μg/kg LPS treatment group).

The LPS challenge significantly decreased duodenal SOD activity, CAT activity, GSH-Px activity, and T-AOC capacity compared to the control group (Figure 3; *P* < 0.05). PLP supplementation significantly restored T-AOC capacity relative to the LPS group (*P* < 0.05) and attenuated the LPS-induced increase in MDA content (*P* < 0.05). However, PLP did not significantly reverse the reductions in SOD, CAT, or GSH-Px activities (*P* > 0.05). The findings indicate that PLP possesses the capacity to mitigate the decrease in T-AOC levels and the elevation in MDA content in the duodenal tissue of broilers induced by LPS.

### 3.7 Effect of PLP on duodenal immunity indexes in LPS-induced broilers

**Figure 4:**
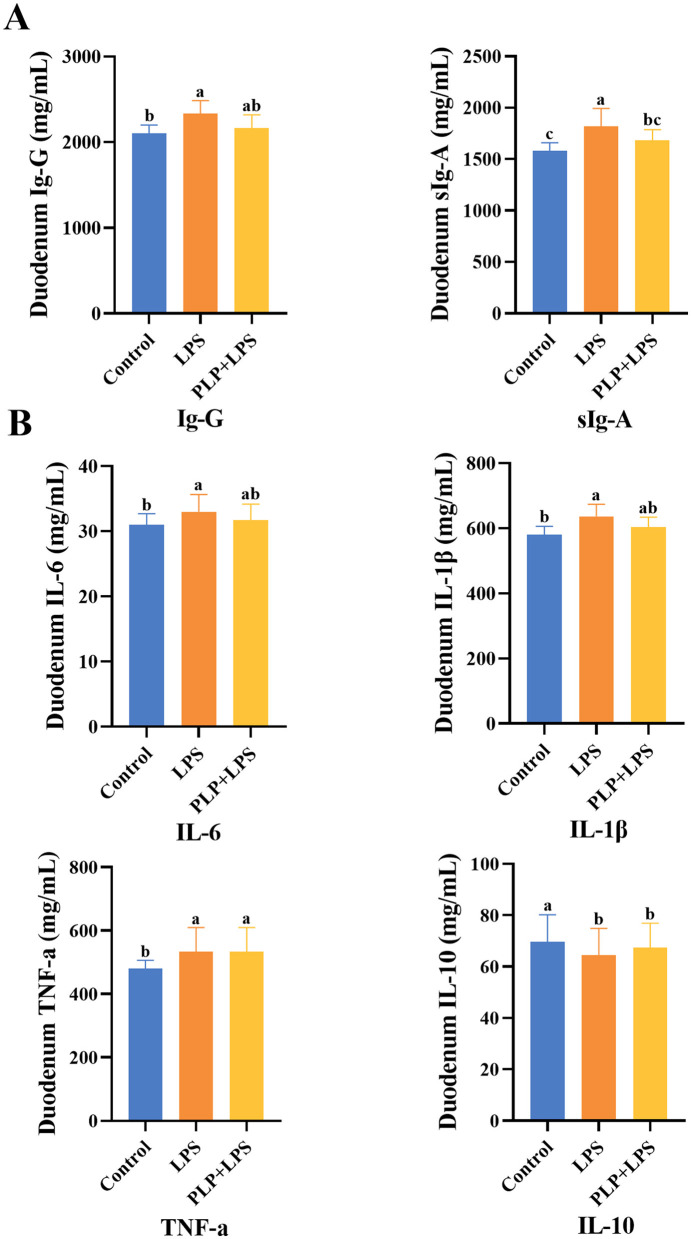
The effect of PLP on duodenal immune indicators and inflammatory factors of LPS challenged broilers. The effect of PLP on duodenal immune indicators **(A)** and inflammatory factors **(B)** of LPS challenged broilers. In the bar chart, the same superscript or no letter means no significant difference (*P* > 0.05), different lowercase letters means significant difference (*P* < 0.05). Control (blank control group); LPS (1,500 μg/kg LPS challenge group); PLP + LPS (200 mg/kg PLP + 1,500 μg/kg LPS treatment group).

The duodenal IgG and sIgA levels exhibited a significant increase in the LPS group compared to the control group (Figure 4A; *P* < 0.05). In contrast, the PLP+LPS group demonstrated a significant reduction in sIgA levels compared to the LPS group (Figure 4A; *P* < 0.05). These results suggest that PLP effectively mitigated the excessive elevation of duodenal sIgA levels in broiler chickens induced by LPS, thereby attenuating the overactivation of the immune system.

### 3.8 Effect of PLP on duodenal inflammatory factors in LPS-induced broilers

The levels of IL-6, IL-1β, and TNF-α in duodenal tissues were significantly elevated in the LPS group compared to the control group (Figure 4B; *P* < 0.05), whereas IL-10 levels were significantly reduced (*P* < 0.05). No significant differences were observed in these cytokines between the PLP+LPS and LPS groups (*P* > 0.05).

### 3.9 Effects of PLP on the morphological structure of duodenum in LPS-induced broilers

**Figure 5:**
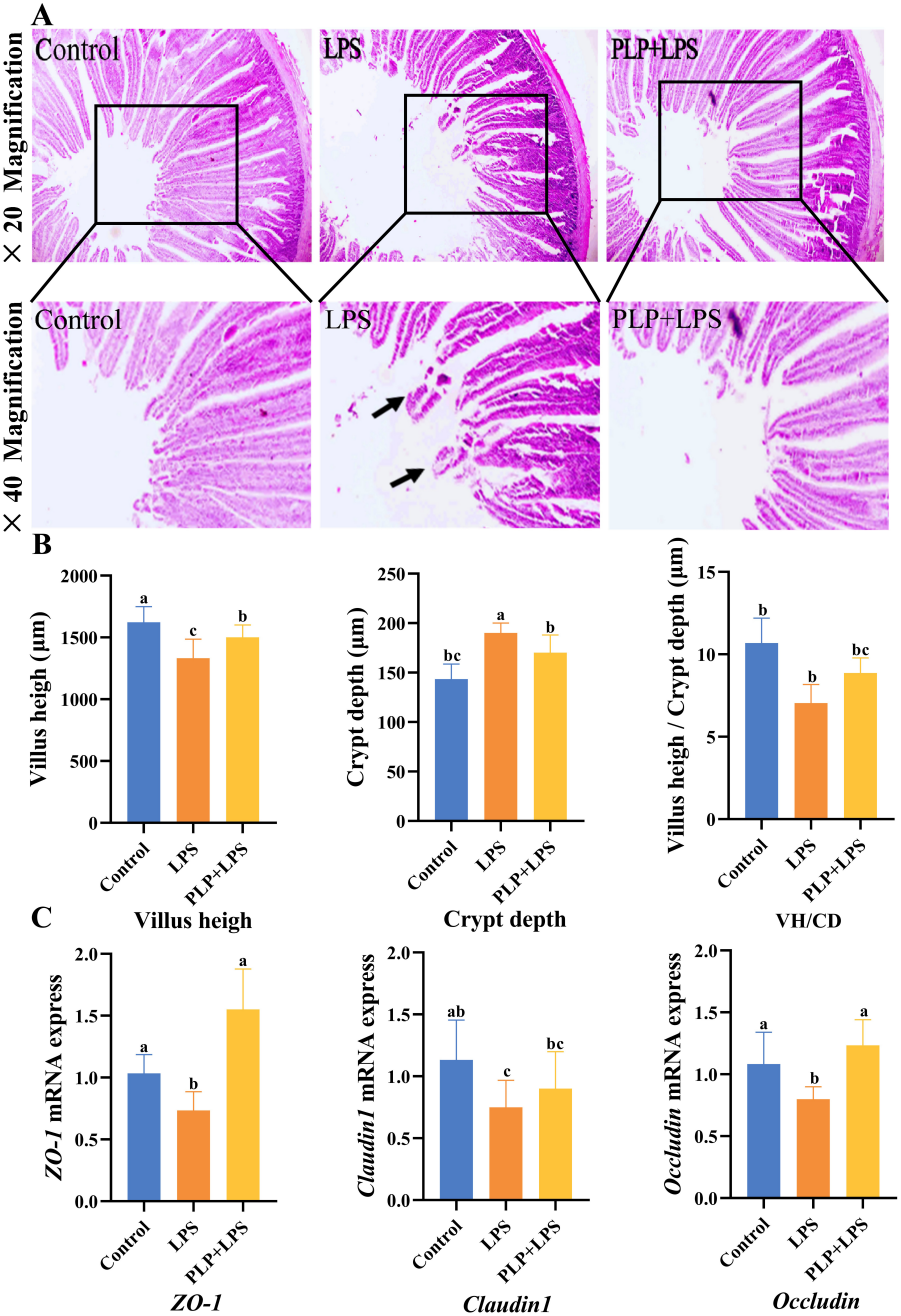
The alleviating effect of PLP on duodenal stress injury in broiler chickens and its impact on the mRNA expression of *ZO-1, Claudin-1* and *Occludin* proteins in the duodenum. Duodenal tissue section (× 20 and × 40). Black arrows indicate damage and shedding of intestinal villi **(A)**. The villus height, crypt depth, and villus height/Crypt depth of duodenum in broiler chickens **(B)**. The effect of LPS stress on the mRNA expression of *ZO-1, Claudin-1* and *Occludin* in the duodenum **(C)**. In the bar chart, the same superscript or no letter means no significant difference (*P* > 0.05), different lowercase letters means significant difference (*P* < 0.05). Control (blank control group); LPS (1,500 μg/kg LPS challenge group); PLP + LPS (200 mg/kg PLP + 1,500 μg/kg LPS treatment group).

Histopathological examination (Figures 5A, B) revealed that LPS challenge induced significant duodenal mucosal injury, featuring villus shortening and epithelial detachment. Quantitative analysis showed the LPS group had significantly reduced VH and increased CD versus controls (*P* < 0.05). PLP supplementation significantly restored VH and reduced CD compared to the LPS group (*P* < 0.05), although the V/C ratio improvement did not reach statistical significance (*P* > 0.05). These findings demonstrate that PLP effectively preserves small intestinal mucosal integrity.

### 3.10 Effect of PLP on duodenal tight junction protein (*ZO-1, Claudin-1*, and *Occludin*) mRNA expression in LPS-challenged broilers

To evaluate PLP's effect on intestinal barrier function, we analyzed duodenal mRNA expression of tight junction proteins (*ZO-1, Claudin-1*, and *Occludin*) by qRT-PCR. As shown in Figure 5C, LPS challenge significantly downregulated *ZO-1, Claudin-1*, and *Occludin* expression compared to controls (*P* < 0.05). PLP supplementation significantly upregulated *ZO-1* and *Occludin* expression relative to the LPS group (*P* < 0.05). These findings indicate that PLP enhances intestinal barrier integrity during inflammatory challenge through modulation of tight junction protein expression.

### 3.11 Intestinal microbiota analysis

**Figure 6:**
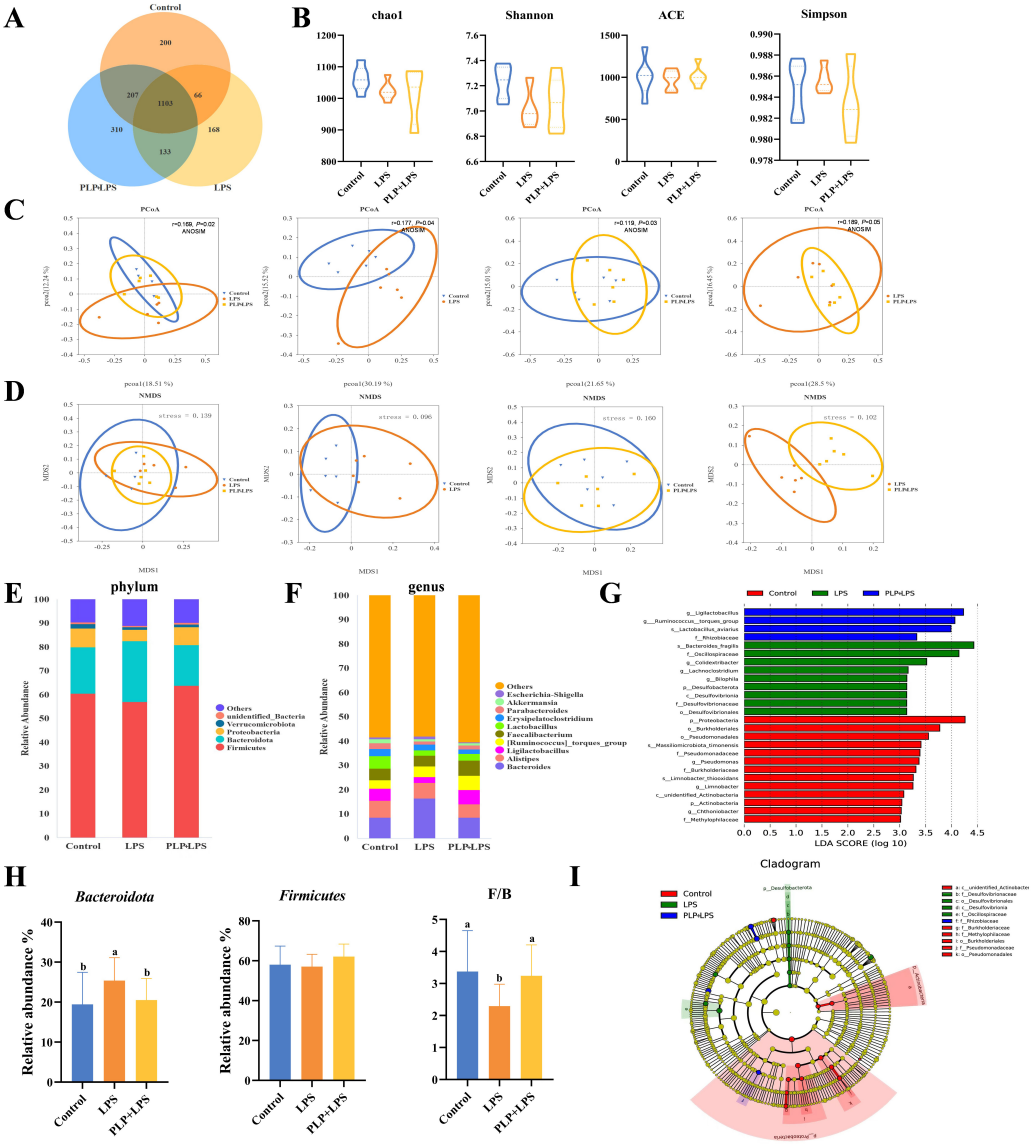
Effect of PLP on the gut microbiota of broilers. Venn diagram of core operational taxonomic units (OTUs) in duodenal content **(A)**. Effects of PLP on the α-diversity of duodenal microbiota. The diversity was evaluated by **(B)** Chao1 index, ACE index, Shannon index, and Simpson index. Beta diversity analysis: principal component analysis based on unweighted-unifrac algorithm **(C)**. NMDS based on bray-curtis algorithm **(D)**. Duodenal bacterial community composition of the control, LPS and PLP + LPS groups at the phylum and genus levels. The different colors of the bars represent different species, and the length of the bars represents the proportion of the species **(E, F)**. Linear discriminant analysis (LDA) effect size (LEfSe) analysis of duodenal microbiota. LDA scores generated for the differentially abundant microbiota (LDA ≥ 3, *P* < 0.05) **(G, I)**. The relative abundance of *Firmicutes, Bacteroidota*, and the ratio of *Firmicutes* to *Bacteroidota* (F/B) in each group based on their relative abundance **(H)**. In the bar chart, the same superscript or no letter means no significant difference (*P* > 0.05), different lowercase letters means significant difference (*P* < 0.05). Control (blank control group); LPS (1,500 μg/kg LPS challenge group); PLP + LPS (200 mg/kg PLP + 1,500 μg/kg LPS treatment group).

Microbiota analysis identified 1,576, 1,470, and 1,753 unique OTUs in the control, LPS, and PLP+LPS groups, respectively, with 1,103 OTUs shared among all groups (Figure 6A). No significant differences in alpha diversity were observed (Figure 6B; *P* > 0.05). Beta diversity analysis revealed significant treatment effects. PCoA based on Unweighted Unifrac distance demonstrated distinct clustering patterns (*P* < 0.05), showing clear separation between LPS and control group, as well as between PLP+LPS and LPS group (Figure 6C). NMDS analysis using Bray-Curtis distance (stress value <0.05) confirmed these findings, indicating that both LPS and PLP+LPS treatments significantly altered the duodenal microbiota composition compared to control (Figure 6D). The results showed that compared with the control group, LPS and PLP+LPS significantly altered the β-diversity of duodenal microbiota. Similarly, there are significant differences between LPS and PLP+LPS.

Classification of gut microorganisms by phylum revealed that *Firmicutes, Bacteroidetes, Verrucomicrobiota*, and *Proteobacteria* comprised the four primary bacteria in the duodenum of broilers, accounting for over 90 percent among all bacteria (Figure 6E). *Bacteroidetes* was the relatively abundant of the duodenal flora, with a relative abundance of approximately 19% in the control group, increasing to 25% in the LPS group, and increase of about 21% in the PLP+LPS group. PLP treatment partially reversed the increase in relative abundance in the LPS group. LPS significantly reduced the ratio of *Firmicutes* to *Bacteroidetes* compared to the other two groups, adding PLP changed this phenomenon (Figure 6H; *P* < 0.05).

Groups of gut microorganisms were classified by genus (Figure 6F). When considering the genus-level analysis, the LPS group exhibited a notably higher relative abundance of *Bacteroides* and *Escherichia-Shigella* compared to the other groups (*P* < 0.05). The addition of PLP significantly reduced the relative abundance of *Bacteroides* and *Escherichia-Shigella* (*P* < 0.05). The relative abundance of *Ligilactobacillus* and *Lactobacillus* decreased significantly (*P* < 0.05) (Supplementary Figure S1). An examination of the distinctively abundant taxa across the three groups uncovered that: *g_Ligilactobacillus, g_Ruminococcus_torques_group, s_Lactobacillus_aviarius* and *f_Rhizobiaceae* were enriched in the PLP+LPS group (*P* < 0.05), while *s_Bacteroides_fragilis, f_Oscillospiraceae, g_Colidextribacter, g_Lachnoclostridium, g_Bilophila, p_Desulfobacterota, c_Desulfovibrionia, f_Desulfovibrionaceae* and *o_Desulfovibrionales* were enriched in the LPS group (*P* < 0.05), and *p_Proteobacteria, o_Burkholderiales, o_Pseudomonadales, s_Massiliomicrobiota_timonensis, f_Pseudomonadaceae, g_Pseudomonas, f_Burkholderiaceae, s_Limnobacter_thiooxidans, g_Limnobacter, c_unidentified_Actinobacteria, p_Actinobacteria, g_Chthoniobacter* and *f_Methylophilaceae* were enriched in the control group (Figures 6G, I; *P* < 0.05).

### 3.12 The spearman correlation analysis

**Figure 7:**
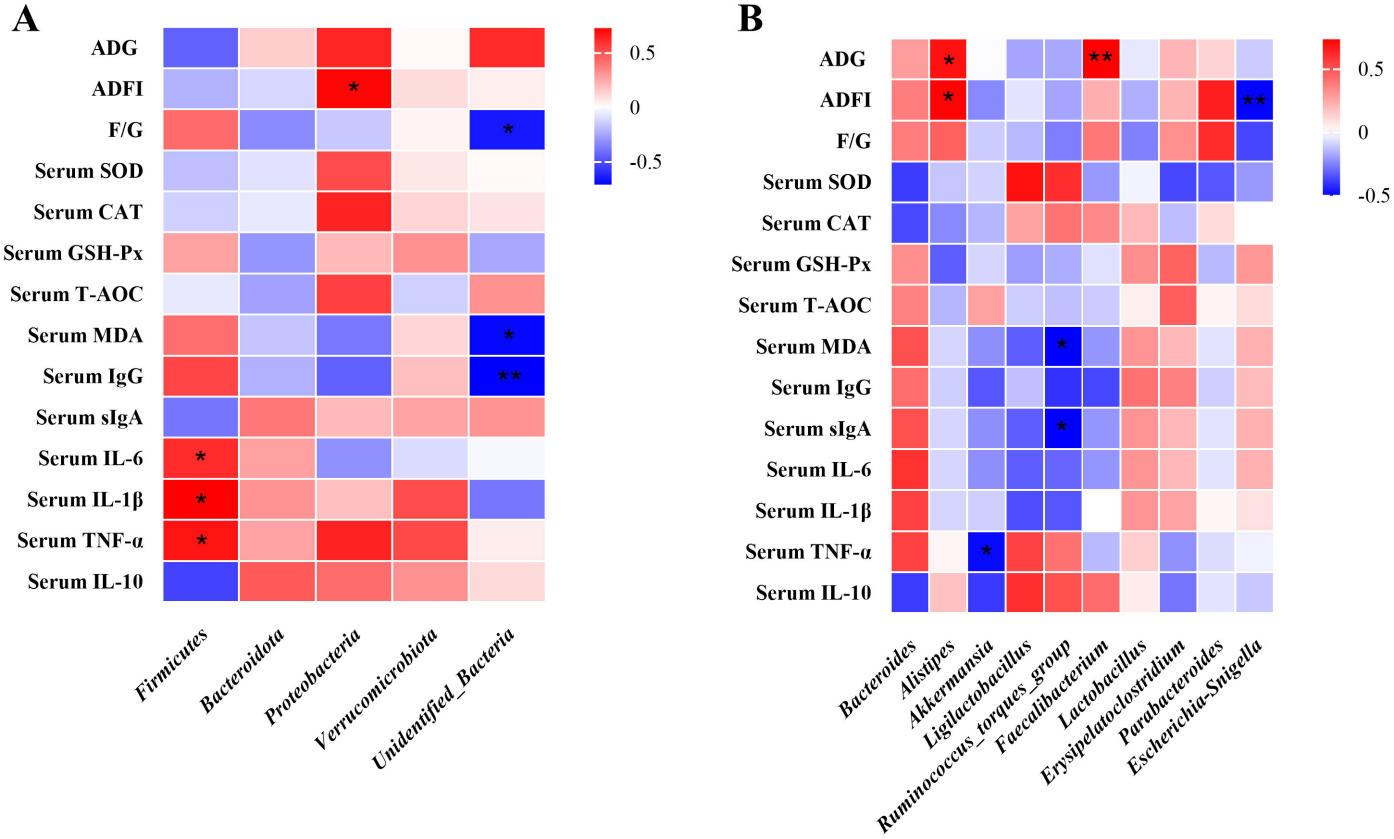
Spearman correlation analysis of broiler intestinal microbiota composition at both phylum and genus levels with growth performance, antioxidant capacity, and immune function parameters. Spearman correlation coefficients of ADG, ADFI and F/G, serum antioxidant capacity, and cytokine concentrations with representative duodenal microbiota at phylum **(A)**, and genus **(B)** level, are represented by color ranging from red (positive correlation) to blue (negative correlation), respectively. * and ** indicates statistically significant difference (*P* < 0.05) and (*P* < 0.01), respectively. BW, body weight; ADG, average daily gain; ADFI, average daily feed intake; T-AOC, total antioxidant capacity; CAT, catalase; SOD, superoxide dismutase; MDA, malondialdehyde; VH, villus height; TNF-α, tumor necrosis factor-α; IL-1β, interleukin-1β.

At the phylum level, the relative abundance of *Firmicutes* showed a significant positive correlation with serum IL-6, IL-1β, and TNF-α levels (*P* < 0.05), while *Proteobacteria* abundance was positively associated with ADFI (*P* < 0.05). Additionally, F/G ratio, serum MDA, and serum IgG concentrations were negatively correlated with the relative abundance of *Unidentified_Bacteria* (*P* < 0.05) (Figure 7A). At the genus level, *Alistipe*s abundance exhibited a positive correlation with both ADG and ADFI (*P* < 0.05). In contrast, *Akkermansia* abundance was negatively associated with serum TNF-α levels (*P* < 0.05). Furthermore, the *Ruminococcus_torques_group* showed a negative correlation with serum MDA and sIgA (*P* < 0.05), whereas *Faecalibacterium* abundance was positively correlated with ADG (*P* < 0.01). Notably, a significant negative correlation was observed between *Alistipes* abundance and ADFI (*P* < 0.01) (Figure 7B).

### 3.13 PICRUSt function analysis

**Figure 8:**
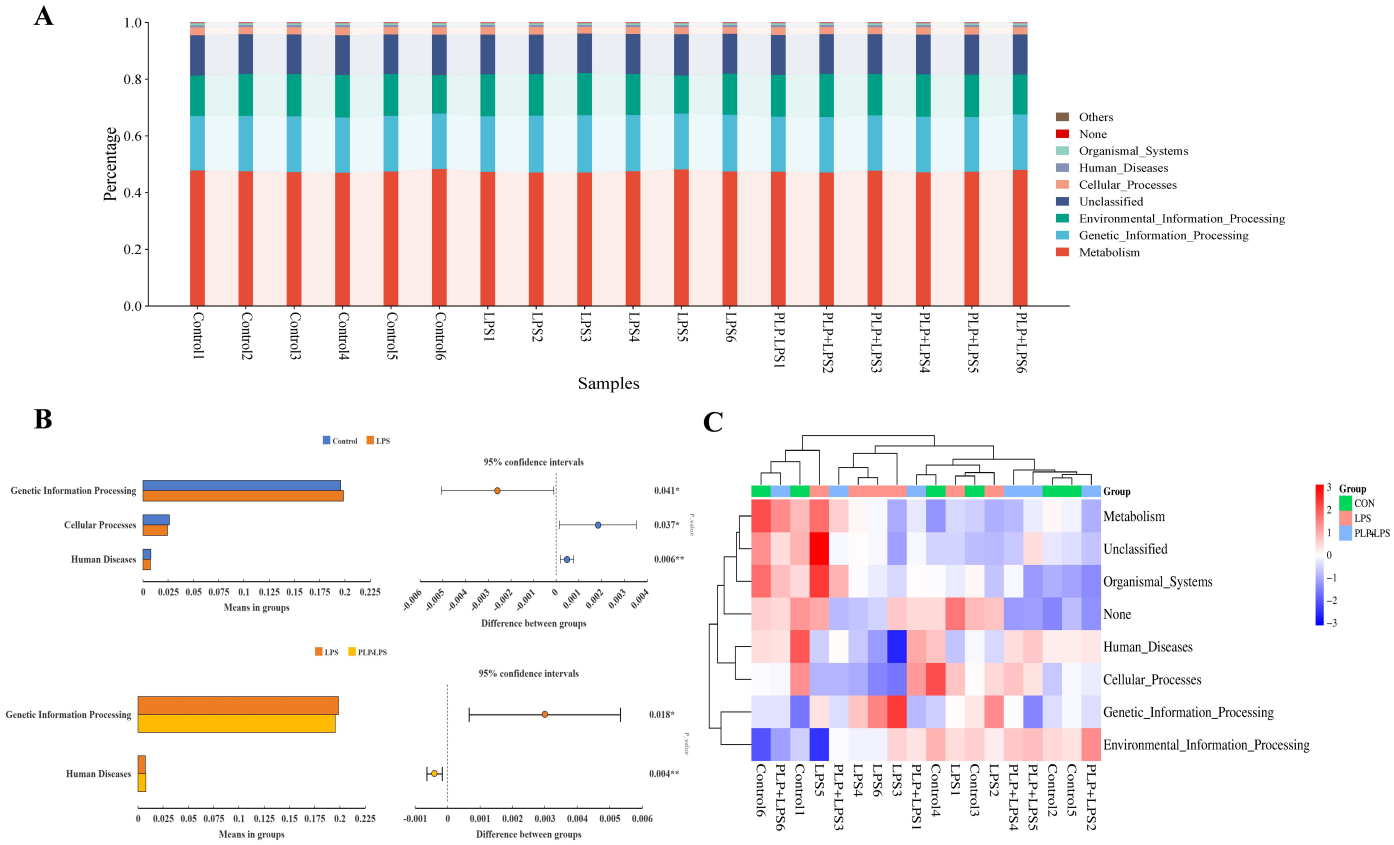
KEGG-based function prediction. **(A)** Relative abundance of relative abundance of functions. The horizontal axis represents the sample name and the vertical axis represents the relative abundance of the predicted function. **(B)** Figure of analysis of KEGG metabolic pathways difference between groups. Abundance proportion of different functions in two groups of samples was shown in the left part of the figure. The difference proportion of functional abundance within the 95% confidence interval was shown in the middle part of the figure and the value on the far right is *P*-values. KEGG, Kyoto encyclopedia of genes and genomes. **(C)** PICRUSt functional abundance clustering heatmap. The horizontal axis represents the sample name, and the vertical axis represents the function name. The redder the color in the graph, the higher the abundance, and the bluer the color, the lower the abundance.

PICRUSt Function Analysis is a KEGG database-based functional prediction beionged to 16S rRNA sequencing data to further reveal the differences in functional genes of intestinal bacteria. Based on the database annotation results, the functional information of the top 7 maximum abundance rankings at level1 annotation level for each sample was selected to generate a functional relative abundance bar stacking plot (Figure 8A). The results of this experiment showed that KEGG pathway was mainly enriched in Metabolism, Genetic_Information_Processing, Environmental_Information_Processing, Cellular_Processes, Human_Diseases, Organismal_Systems, etc. The difference in bacterial functional gene abundance between the control and LPS groups (*P* < 0.05) was mainly related to the KEGG levels of Genetic_Information_Processing, Cellular_Processes and Human_Diseases. While the difference in bacterial functional gene abundance between the LPS and PLP+LPS groups (*P* < 0.05) was mainly related to the KEGG levels of Genetic_Information_Processing and Human_Diseases (Figure 8B). In Figure 8B, the predictive function with the highest relative abundance is the Genetic_Information_Processing. The abundance of the predictive function of Genetic_Information_Processing was significantly higher (*P* < 0.05) in the LPS group compared with the control group, and decreased (*P* < 0.05) with the addition of PLP. The heatmaps were plotted in the results of the functional prediction analysis in the level1 database based on the functional annotations and abundance information of the samples in the KEGG database by selecting the top 7 functions in terms of abundance and their abundance information in each sample (Figure 8C). Similarly, the heatmap shows that the predicted functional abundance of Genetic_Information_Processing in each sample of the LPS group is higher than that of the PLP+LPS group.

## 4 Discussion

Several studies have shown the antioxidant, anti-inflammatory and immunomodulatory properties of PLP (Wang et al., 2014; Park, 2017; Shin et al., 2021). Accumulating evidence suggested that PLP effectively enhances intestinal barrier function (Liu et al., 2020). LPS was widely used to simulate infection by pathogens in broilers, aiming to investigate immune and inflammatory reactions (Zhang et al., 2021; Erinle et al., 2022; He et al., 2022). Despite this, the research pertaining to the influence of PLP on the early growth performance, antioxidant capabilities, immunological function, intestinal health, and microbiota composition of AA white broilers remains scarce. Accordingly, the purpose of this investigation aimed to explore the impacts of PLP on the growth performance, immune organ indices, immune functionality in serum and intestines, as well as the antioxidant capacity of early-stage white-feathered broilers. Additionally, it examined intestinal tissue morphology, expression of tight junction protein genes, and gut flora communities.

Previous research has shown that the incorporation of LPS in the diet of birds results in a reduction in both the ADG and ADFI, accompanied by an increase in the feed-to-gain ratio (Li et al., 2021; He et al., 2022; Wang J. et al., 2023). The inclusion of LPS notably reduced both the ADG and ADFI, subsequently resulting in a higher feed-to-gain ratio and consequently affecting the growth of white-feathered chickens in a negative manner. Conversely, the supplementation the feed with PLP positively influenced the ADG and feed consumption of white-feathered broilers, leading to a decrease in the feed-to-gain ratio. Dietary inclusion of PLP mitigated LPS-induced adverse effects on broiler growth, establishing a theoretical basis for using PLP to promote growth under LPS challenge.

Important immunological organs and tissues in birds include the thymus, spleen, and bursa of Fabricius. Their indices can show how antigenic stimulation affects the organism (Zhou et al., 2019). In the immunocompetent states, thymus and the bursa of Fabricius function as significant immune tissues, essential for the development, differentiation, and maturation of T lymphocytes and B lymphocytes (Zhang et al., 2022; Trujillo Reyes et al., 2024). Furthermore, the spleen is a vital organ for avian immune response, serving as a pivotal factor in producing antibodies and facilitating cellular immunity (Noble et al., 2018). As previously reported, multiple intraperitoneal injections of 1,500 μg/kg BW LPS in Kobo broilers significantly increased their spleen index and showed a trend toward an increase in the bursa index (Wang et al., 2016). The study demonstrated that LPS stimulation elevated the spleen and bursa indices of broilers, which was consistent with the above studies. The splenic tissue hypertrophy induced by LPS is attributed to the immune response triggered by LPS, leading to increased inflammatory cells and compensatory splenic hyperplasia due to lymphocyte proliferation (Du et al., 2023). Notably, the addition of PLP demonstrated a notable inhibitory ramification for proliferation of the bursa of Fabricius proliferation under LPS-induced stress, suggesting at a possible function of PLP in regulating the immune response of the organism.

The oxidative and antioxidant systems in healthy organisms are in dynamic balance. In fact, poultry are highly susceptible to oxidative stress induced by various pathogens and environmental factors (Akbarian et al., 2016; Mishra and Jha, 2019). When poultry are exposed to these stimuli, the organism produces a large amounts of highly reactive free radicals such as ROS. Once free radical concentration surpasses antioxidant capacity, the body's antioxidant balance is disrupted. Excessive ROS destroy biomolecules and generate MDA, resulting in cell death and tissue harm (Xu et al., 2021). Antioxidant enzymes like SOD, CAT, and GSH-Px, maintain the body's oxidative balance and protect it from oxidative damage (Xie T. et al., 2019). T-AOC serves as a marker for free radical scavenging (Ozdemir et al., 2007), and the organism's total antioxidant capacity can be indicated by assessing the level of T-AOC. Our results showed that exposure to LPS decreased serum SOD, CAT and T-AOC activities and decreased duodenal SOD, CAT, GSH-Px and T-AOC activities while increasing MDA concentration in broilers. This observation suggests that LPS appears to upset the oxidative balance in broilers (Zheng et al., 2016; Chen et al., 2022; Wang S. et al., 2023). It can decrease the organism's antioxidant capacity and the scavenging ability of ROS by antioxidant enzymes, ultimately causing an elevation in ROS content and subsequent oxidative damage to cell tissues. Notably, PLP mitigated the LPS-induced decline in serum T-AOC and SOD activities, improve T-AOC activity in the duodenum, and reduce MDA levels. The improvement is likely due to *Phellinus linteus* extracts' antioxidant properties, enhancing GSH-Px and CAT while decreasing MDA (Zuo et al., 2021; Luo et al., 2021). These experimental results suggest that PLP may protect tissues from lipid over-oxidation by reducing ROS generation, lowering MDA levels, and increasing T-AOC and antioxidant enzyme activities. In summary, PLP mitigates LPS-induced oxidative stress in broiler chickens, restoring redox balance and lessening oxidative damage.

The humoral immune response is effective in mitigating most of the effects of bacteria and viruses on the body (Burton and Maini, 2021), and serum levels of IgG can be used to assess the body's humoral immunity (Liu M. et al., 2023). IgG is pivotal in preventing bacterial and viral invasion and neutralizing toxins (Wei et al., 2022). Based on the research, a significant increase in IgG level in body fluids was observed in broilers injected with LPS solution, which suggests that intraperitoneal injection of LPS induces an immune response in body fluids, leading to abnormal immune hyperfunction. In addition, PLP significantly reduced the LPS-induced elevation of IgG levels in body fluids and alleviated the hyperactivation of the immune system. Intestinal sIgA serves as the initial line of defense against harmful toxins, antigens, and microorganisms invading the intestinal barrier (Mantis et al., 2011). The administration of LPS may potentially compromise this protective mechanism. Lessard et al. studied that LPS tapping increased the intestinal sIgA levels in piglets (Lessard et al., 2009), and in the present trial, LPS stimulation significantly increased the duodenal sIgA levels in broiler chicks, which was improved by the addition of dietary PLP, suggesting that PLP inhibited sIgA secretion, ameliorated LPS-induced intestinal mucosal damage, and attenuated intestinal microbial irritation of the intestinal mucosa.

The secretion of pro-inflammatory cytokines (IL-1β, IL-6, TNF-α) in vivo elicits cellular and tissue responses when the organism is stimulated by pathogenic bacteria or non-pathogenic factors (Wu et al., 2022; Wang et al., 2025). LPS promotes rapid production of inflammatory factors in the body and rapidly activates the immune function in broilers (Qiao et al., 2022). Due to the excessive production and duration of these cytokines, the original nutrients in the organism are diverted to the immune system to maintain immune homeostasis, leading to hypophagia and growth impairment (Samy et al., 2016; Hidayat et al., 2020). This could be a reason for the decrease in growth performance of broiler chicks after LPS-induced. This results demonstrate that PLP inhibited serum levels of IL-6, prevented an elevation of serum levels of IL-6, IL-1β, TNF-α, and IL-10, and elevated duodenal levels of IL-10 in comparison to the control group. The results suggested that dietary inclusion of PLP can effectively reduce the organism and intestinal damage caused by LPS, and helped to reduce intestinal damage and maintain mucosal integrity.

The gut primarily digests and absorbs nutrients, while also serving as the body's largest immune and endocrine organ (Cannon et al., 2011). Small intestinal VH, CD and V/C are indicators of intestinal digestibility and mucosal integrity (Qiao et al., 2022). In the examination, we determined that LPS decreased duodenal VH and V/C. However, dietary inclusion of PLP increased duodenal VH and V/C values, showing a protective effect on duodenal intestinal structure. The expanded absorption area in the small intestine boosted digestion, absorption, and nutrient utilization, enhancing overall efficiency. Reducing the damage of LPS on the morphology of the duodenum in white feather broilers, thus improving the integrity of the duodenal mucosa. These findings are consistent with Liu's conclusions about PLP's effects on intestinal morphology and protection (Liu et al., 2020).

Gene expression levels of tight junction proteins (*ZO-1, Claudin-1* and *Occludin*) in intestinal mucosal epithelial cells reflect intestinal permeability and mucosal barrier integrity (Buckley and Turner, 2018). *ZO* proteins, composing of *ZO-1, ZO-2*, and *ZO-3*, function as cytoplasmic adaptor proteins, anchoring tight junction complexes to the actin cytoskeleton. Meanwhile, claudins, a family of membrane proteins, function as the structural backbone of tight junctions (Nguyen et al., 2024). *Occludin*, a critical structural component of tight junctions, is another integral membrane protein (Cui et al., 2021). It interacts with *Claudins* and other tight junction proteins to form a complex network of protein-protein interactions (Feldman et al., 2005). The necessity for a highly discriminatory intestinal barrier is heightened by the intestinal lining's constant interaction with luminal bacteria, bacterial metabolites, and dietary antigens. Tight junction proteins constitute the gut's physical barrier, which regulates intestinal permeability and is pivotal in preserving the integrity of the gastrointestinal tract. Intraperitoneal injection of LPS has been shown to reduce intestinal tight junction proteins (He et al., 2020; Hu et al., 2020; Xu et al., 2020). As shown in the experiments, PLP increased the reduction of mRNA expression levels of duodenal *ZO-1, Claudin-1* and *Occludin* induced by LPS. A growing body of evidence from a number of studies indicates that polysaccharides derived from plants can attenuate LPS appears to attenuate intestinal injury by maintaining small intestinal mucosal integrity and increasing tight junction protein gene expression and improving permeability.

The gut microbiota plays a crucial role in nutrient digestion, gut barrier maintenance, and immune regulation (Yadav and Jha, 2019). It actively interacts with the intestinal barrier to maintain its integrity (Paone and Cani, 2020). *Phellinus linteus* is a rare medicinal mushroom (Wang et al., 2022). Like *Coriolus versicolor, Ganoderma lucidum*, and *Agaricus bisporus*, it contains fermentable polysaccharides that effectively modulate the gut microbiota with minimal adverse effects (Li et al., 2016; Matijašević et al., 2016; Tian et al., 2018). Beyond mushrooms, natural products such as *Abies alba* water extract also exhibit prebiotic effects on the gut microbiota (Stojanov et al., 2021). Previous research has shown that PLP alters gut microbiota composition (Liu T. et al., 2023; Qin et al., 2023). In this study, PCoA revealed significant separation between the microbial communities of the control vs. LPS group, control vs. PLP group, and PLP vs. PLP+LPS group, indicating that dietary PLP reshapes duodenal microbiota structure. The PLP treatment significantly enhanced the β-diversity of intestinal microbiota, thereby promoting the recovery of gut health.

Analysis at the phylum level demonstrated that *Firmicutes* and *Bacteroidota* constituted the majority of duodenal microbiota in broilers, consistent with prior studies (Liu et al., 2021; Qiao et al., 2022). PLP supplementation significantly increased the F/B ratio, an effect similar to that observed with resveratrol (Cai et al., 2020), and specifically elevated *Firmicutes* abundance, a phylum associated with enhanced nutrient metabolism and anti-inflammatory activity (Wang et al., 2021). Spearman analysis further showed a positive correlation between *Firmicutes* abundance and pro-inflammatory cytokines (IL-6, IL-1β, TNF-α). This correlation arises from the upregulation of these cytokines due to the inflammatory response occurring in the intestine, ultimately resulting in alterations to the gut microbiota composition (Rabiei et al., 2019). These findings highlight duodenal microbiota modulation as a potential mechanism through which PLP exerts its protective effects against LPS challenge.

At the genus level, PLP supplementation significantly modulated gut microbiota composition by increasing beneficial genera while suppressing pathogenic species. We observed substantial enrichment of *Ligilactobacillus*, which enhances intestinal barrier function through upregulation of tight junction proteins *ZO-1* and *occludin*, thereby reducing intestinal permeability (Chuandong et al., 2024; Zang et al., 2024). The mucin-degrading genus *Akkermansia* also showed significant expansion, known to maintain microbial homeostasis by competitively inhibiting pathogenic *Bacteroides* (Zhang et al., 2024). Importantly, PLP supplementation markedly reduced the abundance of pathogenic *Escherichia-Shigella*, a well-characterized enteropathogen associated with bacillary dysentery and intestinal inflammation that is frequently enriched in inflammatory bowel disease (IBD) patients (Maurelli et al., 1998; de Paiva et al., 2016). These coordinated microbial changes were particularly evident in the PLP+LPS group compared to LPS controls, demonstrating PLP's ability to counteract dysbiosis. The combined promotion of beneficial commensals and suppression of pathogens suggests PLP supplementation can ameliorate intestinal immune stress through targeted microbial modulation. These findings support the potential of dietary PLP to enhance intestinal health in broilers by favorably reshaping microbial communities to reduce enteric disease risk.

Notably, *Akkermansia* and *Lactobacillus* are common genera in probiotic formulations and have shown promising results in reducing intestinal permeability, enhancing intestinal barrier function, ameliorating inflammation, boosting immunity, and maintaining gut microbiota balance (DiMattia et al., 2024; Liu et al., 2024). Research has indicated that *Faecalibacterium* facilitates the production of anti-inflammatory factors like IL-10 (Sun et al., 2022). This experiment is consistent with its research results. Spearman correlation analysis revealed that the abundance of *Faecalibacterium* and *Alistipes* were positively correlated with growth performance in broilers. The observed correlation might stem from the capacity of *Faecalibacterium* and *Alistipes* to promote intestinal mucus production and optimize gut function (Lin et al., 2023; He and Dong, 2023; Wang et al., 2024), which creates a favorable environment for growth. These findings suggest that PLP supplementation may induce beneficial modifications in the gut microbiota composition. Such modifications could potentially lead to improved intestinal morphology and enhanced nutrient absorption capacity in broilers, thereby promoting growth performance.

*Parabacteroides* possesses the capability to modulate the immune response of the host, achieved through enhancing intestinal levels of sIgA and butyric acid (Guo et al., 2021). This also explains why the addition of PLP increased LPS-induced duodenal slgA levels in broilers in this study. By disrupting the balance of gut microbiota, *Bacteroides* and *Escherichia-Shigella* fostered the multiplication of pathogenic bacteria, subsequently triggering the production or accumulation of harmful substances (Deng et al., 2023). The correlation analysis showed that *Bacteroides* were positively correlated with serum inflammatory cytokines TNF-α, IL-1β, and IL-6. However, beneficial bacteria such as *Akkermansia, Faecalibacterium*, and *Parabacteroides* have been demonstrated to have beneficial effects on intestinal inflammation and enhance intestinal barrier integrity. They can alleviate the inflammatory response and intestinal barrier damage caused by LPS-induced. The study's functional predictions revealed that the Genetic_Information_Processing function exhibited the highest relative abundance. However, the specific manner in which it operates within the chicken gut microbiota remains uncertain and necessitates additional follow-up studies for clarification.

## 5 Conclusion

In summary, PLP demonstrates the ability to counteract LPS-induced injury by enhancing production performance, alleviating oxidative stress, mitigating pathological damage, and bolstering immune function. Our findings suggest that PLP exerts a mitigating effect on LPS-induced injury in broilers by means of its antioxidants properties, restoration of the intestinal barrier, and modulation of gut microbiota structure. Consequently, PLP represents a promising feed additive for implementation in poultry production, as it has the potential to enhance growth performance, alleviate oxidative stress, boost immune function, and safeguard against potential exogenous pathogen intrusion.

## Data Availability

The data presented in the study are deposited in online repositories. The data can be found here: NCBI BioProject number PRJNA1262155, available at: https://www.ncbi.nlm.nih.gov/sra/PRJNA1262155.
