# Supplementary figures and images for: Modulating effects of Phellinus linteus polysaccharides on antioxidant capacity, immune function, intestinal function and microbiota in lipopolysaccharide-challenged broilers
Source: Front Microbiol. 2025 May 27;16:1570370. doi: 10.3389/fmicb.2025.1570370 (PMC12150300; doi:10.3389/fmicb.2025.1570370)

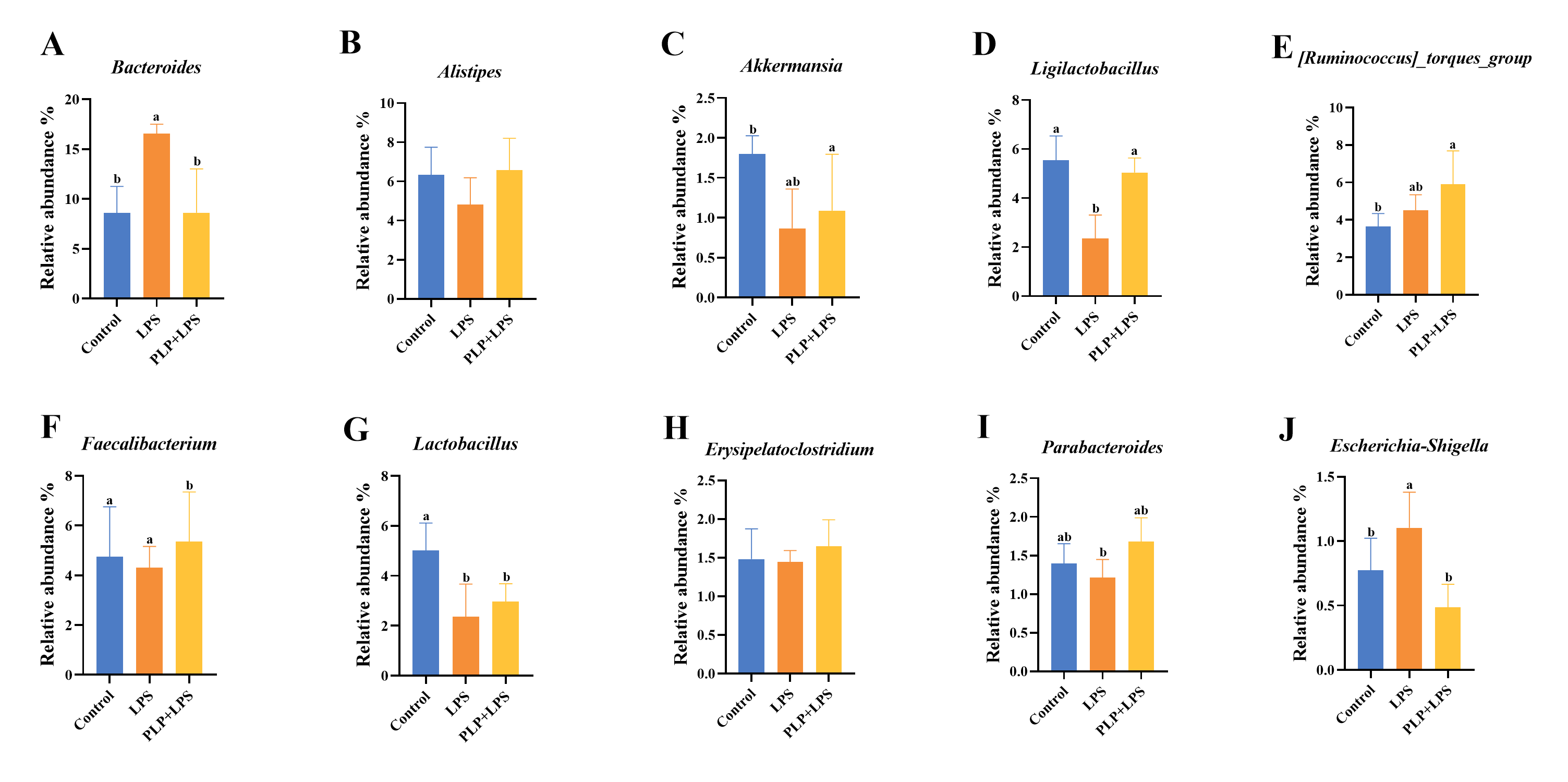

Supplement: Supplementary file 1 [file Image_1.tif]
